# Larvicidal Efficacy of Ozone and Ultrasound on *Angiostrongylus cantonensis* (Rat Lungworm) Third-Stage Larvae

**DOI:** 10.3390/foods11070953

**Published:** 2022-03-25

**Authors:** Argon Steel, Matthew S. Platz, Alyssa-Jade Riglos, Billie Jean Garcia, John Jacob, Susan I. Jarvi

**Affiliations:** 1 Department of Pharmaceutical Sciences, Daniel K. Inouye College of Pharmacy, University of Hawai’i at Hilo, 200 West Kawili St., Hilo, HI 96720, USA; argon@hawaii.edu (A.S.); johnj7@hawaii.edu (J.J.); 2 Department of Chemistry, University of Hawai’i at Hilo, 200 West Kawili St., Hilo, HI 96720, USA; mplatz@hawaii.edu (M.S.P.); ajriglos@hawaii.edu (A.-J.R.); bjarcia@hawaii.edu (B.J.G.)

**Keywords:** *Angiostrongylus cantonensis*, angiostrongyliasis, rat lungworm, food safety, ozone, ultrasound

## Abstract

The parasitic nematode *Angiostrongylus cantonensis* (rat lungworm) is the leading cause of human eosinophilic meningitis worldwide. Most human infections occur through the accidental consumption of *A. cantonensis* hidden within produce as infectious third-stage larvae (L3), yet little research has been published addressing possible methods to mitigate this means of transmission. Here, we describe our tests of ozone gas—an oxidizing agent—and ultrasound, both used for disinfection of food and municipal water supplies and in industrial cleaning. We found that exposure to ozone, produced using two different commercially available ozone generators over varying durations of time and concentrations, was capable of achieving 100% larval mortality. In addition, we evaluated the impact of different sound frequencies on *A. cantonensis* L3 survival using two different commercially available ultrasonic cleaners, and found that 60 s of 40 kHz produced 46% mortality within 2 h. The combined use of ultrasound and ozone gas simultaneously resulted in a minimum of 89% normalized mean percent mortality within 2 h of treatment. Our study suggests that both ozone and ultrasound show high larvicidal efficacy, both independently and together, and thus show promise as methods for reducing the risk of rat lungworm infection via accidental consumption.

## 1. Introduction

The nematode *Angiostrongylus cantonensis*—the rat lungworm—is a zoonotic parasite that causes angiostrongyliasis or rat lungworm disease, and is the leading cause of human eosinophilic meningitis worldwide [1,2]. The *A. cantonensis* life cycle involves its development from the first (L1) to the third (L3) larval stages within its obligatory, intermediate hosts—mollusks. This L3 stage is infective to its definitive host, rats (*Rattus* spp.), in which it grows and reproduces, as well as to such accidental hosts as humans, dogs, horses, and other species. Humans are considered as a “dead-end” host, i.e., *A. cantonensis* has not been documented to reproduce in humans, but instead remains in the central nervous system or can move to the eye chamber (causing ocular angiostrongyliasis). In either location, larvae remain motile in tissues until parasite death. [3] Angiostrongyliasis in humans can range from relatively mild and self-resolving (headache, stiff neck, vomiting, and nausea) to severe (paralysis, blindness, and fatal encephalitic meningitis), with characteristic eosinophilia of the peripheral blood and cerebrospinal fluid [4]. While first identified in China [5], and originally endemic in Asia, *A. cantonensis* has been increasingly detected throughout the Americas [6]—particularly Hawai‘i Island, which has become a hotspot for rat lungworm disease in the United States [7,8,9,10].

Most human infections are presumed to be through either the consumption of inadequately cooked foods containing infective *A. cantonensis* larvae, through contaminated water [11] or, as in the USA, from the accidental ingestion of infected mollusks, typically hidden in uncooked vegetables and fruits [6]. Despite the importance of this mode of infection, relatively few papers have investigated treatments aimed at reducing the risk of food being a vehicle for *A. cantonensis* transmission [12,13,14]. We recently reported on the results of a series of in vitro tests in which we compared the efficacy of a variety of solutions for killing infectious-stage (L3) *A. cantonensis* larvae [15]. The solutions tested included common household products, consumer vegetable washes, and agricultural crop washes. While we found little efficacy in our tests of consumer-grade fruit and vegetable washes, botanical extracts such as ginger or garlic, or acidic solutions such as vinegar, we did find larvicidal potential in our tests of alkaline solutions, surfactants such as dodecylbenzene sulfonic acid and, notably, oxidizers such as bleach and chlorine dioxide.

Ozone gas is also a powerful oxidizer with significant disinfectant properties. Extensively applied to drinking water and wastewater treatment, ozone has been recognized as a GRAS (generally recognized as safe) substance by the Food and Drug Administration since 1997 [16]. In the food industry, ozone is used to treat equipment, packaging materials, and foods. Ozone has been shown to work well against a broad array of food spoilage and pathogenic microorganisms, including bacterial cells and spores, parasites, viruses, and fungi [17]. Significantly, ozone has shown particular efficacy in the post-harvest treatment of fresh produce. For example, Kim et al. found that bubbling ozone through shredded lettuce in water reduced the concentration of a common lettuce spoilage bacterium by 1.9 log CFU/g. Bialka and Demirci [18] found that treating blueberries with gaseous ozone resulted in a 2.2 log reduction of *Escherichia coli* O157:H7, while apples treated with aqueous ozone showed a 1–3.7 log reduction in the same pathogen [19]. Ozone also has the advantage over other oxidizers—such as chlorine—of decomposing rapidly to molecular oxygen without leaving a residue. Nevertheless, few studies have examined the effect of ozone on nematodes or other helminths [20,21,22,23], and no studies have reported its effect on *A. cantonensis*.

A number of experiments with ozone have shown that its efficacy in reducing the number of foodborne pathogens is dependent upon accessibility to the microorganism; that is, for pathogen reduction to occur, ozone must be delivered directly to the pathogen using stirring, pumping, bubbling, sonication, abrasion, or pressure washing [17]. Among such methods, we were particularly interested in sonication, as it has been reported to be effective in reducing the number of microorganisms in general, used alone for water sanitation [24,25], or to enhance fruit and vegetable storage [26], as well as in conjunction with ozone [27]—and specifically against nematodes [28]. Sonication, otherwise known as ultrasound, is the production of acoustic waves, generally by the vibration of piezoelectric transducers. Based on frequency, ultrasound can be classified into three main categories: power ultrasound (20–100 kHz), high-frequency ultrasound (100 kHz–1 MHz), and diagnostic ultrasound (1–500 MHz) [26]. Our experiments used power ultrasound, as its lower frequencies of 20–100 kHz are used to generate intense shearing forces in liquids to produce large cavitation bubbles possessing extreme pressure and high temperatures [29]. These forces are thought to damage cell walls and cytoplasmic membranes and produce free radicals that enter the cell and react with the interior components to destroy them [30].

The application of both ozone and sonication to food protection is already recognized commercially, with several devices currently available for purchase that utilize these technologies and are specifically sold as “fruit and vegetable cleaners”. These devices vary in size from those marketed for household use up to those intended for commercial kitchens. However, to our knowledge, this is the first investigation undertaken into the use of ozone and sonication specifically against the medically important helminth *A. cantonensis*; as such, it demonstrates a possible means of reducing the risk of infection from this debilitating parasite.

## 2. Materials and Methods

### 2.1. Gastropod Collection and Larval Preparation

Due to its high infection rate—particularly on the east side of Hawai’i Island [31,32,33]—the semislug *Parmarion martensi* was collected from the Hilo and Puna districts of Hawai’i Island as a source of *A. cantonensis* L3 for this study. Slugs were collected from June to December 2020 and drowned in 50 mL of tap water in Falcon^®^ tubes for a minimum of 60 h, after which the slugs were removed, and the bottom 15 mL from each tube was pipetted into a culture plate (60 mm × 15 mm) [11,15]. The chlorine content of tap water was determined to be <10 ppm using Precision Chlorine Test Paper strips (Bartovation, LLC, Queens, NY, USA), and the pH of the tap water was measured at 8.16 (Corning Pinnacle 530 pH meter, Corning, NY, USA). Larvae from culture plates that exhibited the “S-” and “Q”-like swimming movements attributed to *A. cantonensis* L3 [34] were visualized by 10×–40× microscopy (Leica S9 D and Wild Heerbrugg M4A APO), and pooled by pipette into a single large Petri plate containing approximately 2 mL of tap water. Additional washes (up to 3×) were carried out as necessary to thoroughly separate larvae from debris.

Each treatment run included at least eight tubes of 50 larvae each: one tube of killed untreated, three tubes of live untreated, one tube of killed treated, and three tubes of live treated. Live larvae for each treatment run were prepared by adding 50 live larvae into 20 µL of tap water in three 1.5 mL microcentrifuge tubes. Live untreated larvae served as negative controls. Killed larvae served as positive controls for the propidium iodide assay [35], and were prepared by transferring 200 third-stage larvae into 100 µL of tap water and adding 900 µL of 100% methanol (BDH; VWR International, Radnor, PA, USA), followed by freezing at −80 °C for a minimum of 24 h [35,36]. Tubes of killed larvae controls were thawed and poured into small glass Petri dishes containing 10 mL of tap water, and the methanol was allowed to evaporate for one hour with gentle rocking. Killed control larvae (n = 50) were then transferred into two 1.5 mL microcentrifuge tubes per treatment run (one treated, one untreated).

### 2.2. Ozone Exposure

One killed control tube and three live larvae tubes were exposed to ozone gas under two systems: ozone-suffused tap water, and ozone gas bubbled into water. The latter method was repeated using two different ozone generators. All tests were carried out at room temperature (approximately 20 °C). The concentration of ozone dissolved in water by each ozone generator was determined using the indigo colorimetric method [37]. This method relies upon the decolorization of indigo trisulfonate (Aldrich Chemical Co., Inc., Saint Louis, MO, USA) and measurements of changes in color at 600 nm. The indigo molecule contains a carbon–carbon double bond that is cleaved by ozone with a very large reaction rate constant [38]. To measure residual ozone during the reaction, stock solution (1 g of NaH_2_PO_4_ plus 1 mL of concentrated H_3_PO_4_; zero potassium indigo trisulfonate) was used as a blank before monitoring the absorbance of the reaction mixture.

### 2.3. Cashido 10 Second Machine Using Faucet Delivery

The Cashido “10 Second Machine” (Cashido Corp., Toufen City, Taiwan) is a consumer-grade generator that attaches to a proprietary faucet adapter designed to suffuse ozone gas through tap water. To evaluate the efficacy of this ozone generator, we prepared sets of four 50 mL centrifuge tubes per treatment. Each set of tubes consisted of one control tube of 50 pre-killed L3 and three tubes of 50 live larvae. Treatment consisted of filling each set of four tubes with ozone-suffused water (taking care to avoid loss of larvae from overflow), immediately capping after filling and incubating larvae in ozonated water for 5 min. This treatment was repeated on additional sets of tubes to gauge the effects of more prolonged exposure, as follows: with the initial treatment followed by centrifugation for 5 min at 7000 rpm, water was then pulled off the top of each tube, leaving larvae in 10 mL of water at the bottom of each tube; then, the tubes were refilled with ozonated water and incubated again for 5 min. Thus, each set of four tubes had newly ozonated water added either one, three, or six times to expose each set of larvae to ozone-suffused water for 5, 15, and 30 min, respectively (excluding the time in centrifugation). Exposure to this ozonated water, at any duration, showed no effect on *A. cantonensis* mortality (data not shown).

### 2.4. Cashido Using Direct Delivery of Ozone Gas

The Cashido ozone generator is designed to supply ozone to water running through a faucet (described above). However, the minimal modification of attaching an aquarium air pump (SOBO SB-348, Zhongshan Songbao Electric Co., Ltd., Zhongshan City, China) to the generator air input allowed us to use the Cashido to inject ozone gas directly into water containing *A. cantonensis* L3. We measured the ozone injected by the Cashido into the water as having a concentration of approximately 1.5 mg/L. Because airflow through the generator was insufficient to force ozone through an air stone, the ozone was injected into the water through the cut-off tip of a 5 mL polyethylene transfer pipet (#1022-2500, USA Scientific, Ocala, FL, USA). To minimize operator exposure to ozone, we constructed and ran most tests within an ozone chamber made from a sealable, clear plastic container (Sistema KLIP IT^®^ 1332–900 mL Cracker Container, Sistema Plastics, New Zealand). Immediately before testing, 50 L3 were added to each tube: one tube of 50 dead and three tubes of 50 live L3 per treatment (thus allowing us to examine the effects of treatment in triplicate); then, each tube of L3 was placed inside the chamber and exposed to ozone, with excess ozone from the chamber passed into a canister of manganese dioxide (Ozone Destruct System, Promolife, AR, USA). Initial tests were carried out in 15 mL Falcon tubes filled with 5 mL of tap water for durations of 3, 5, 10, and 15 min, and then repeated in 50 mL Falcon tubes filled with 15 mL of water for 5 min.

### 2.5. Enaly OZX-300 Using Direct Gas Delivery

Based on the results from modifying the Cashido ozone generator to bubble ozone through a hose, for comparison, we tested another commercially available ozone generator—the Enaly OZX-300 (Enaly, Shanghai, China). Like the Cashido, the Enaly generates ozone from air (rather than pure oxygen), but unlike the Cashido, the Enaly has a built-in pump, producing greater airflow (rated by the manufacturer at 2 L/min). Given the higher airflow, we compared the effects on L3 mortality from bubbling ozone into water through an air stone diffuser. The Enaly also differs from the Cashido in having an adjustable ozone output, which we measured as generating ozone at a concentration ranging from approximately 4.5 to 7.9 mg/L. L3 were exposed to Enaly ozone at three settings of the dial: turned fully counterclockwise (minimum output), turned halfway (medium output), and turned fully clockwise (maximum output). All tests were for 5 min, with an additional test of 3 min at maximum output.

### 2.6. Ultrasonic Cleaners

Two models of ultrasonic cleaners were evaluated for their efficacy in killing *A. cantonensis* L3: the Branson Bransonic^®^1510R-MT (Emerson Electric Co., St. Louis, MO, USA), and Fisherbrand^®^ FB-11201 (Elma Schmidbauer GmbH, Singen, Germany). The Branson model operates at a fixed frequency of 40 kHz, while the Fisherbrand can switch between 37 kHz and 80 kHz. Thus, larvae were subjected to ultrasound at 37 kHz, 40 kHz, and 80 kHz. In both machines, the effects of ultrasound were tested for durations of 30 s, 1 min, and 2 min with 50 larvae in 1 mL of tap water per tube (four tubes), including one tube of dead control L3 and three tubes of 50 live L3 per treatment.

### 2.7. Ozone with Ultrasound

We investigated larval mortality in response to the combined application of ozone and ultrasound, using the Branson 1510 ultrasonic cleaner at 40 kHz and the Enaly ozone generator at minimum, medium, and maximum output settings (as described above). Ozone generated by the Enaly was bubbled through an air stone into 15 mL of tap water in 50 mL Falcon tubes containing 50 L3—one tube of dead and three tubes of live L3 per treatment—all for 5 min, while the tubes were simultaneously immersed into the Branson bath and subjected to ultrasound for 1 min.

### 2.8. Propidium Iodide Death Assay

The mortality of *A. cantonensis* L3 in response to exposure to ozone and ultrasound was evaluated using a propidium-iodide-based death assay [15,35]. Briefly, after each treatment of either ozone or ultrasound (or both), larvae were transferred from Falcon tubes into 35 mm × 10 mm glass culture plates for counting into individual wells of a black, transparent-bottom view plate (PerkinElmer, Cat#384, Waltham, MA, USA). Twenty microliters of a 1.25% solution of propidium iodide (PI) (Biotium Inc., Cat#40017, Freemont, CA, USA) in water was added to each well along with sufficient additional water to reach a final volume of 120 µL in each well, then gently mixed on a variable-speed rocker for 1 h. Plates were imaged using an Operetta high-content imaging system (PerkinElmer Life Sciences, Boston, MA, USA), and images were collected using a 20× long-working-distance lens with bright-field, propidium iodide (535 nm excitation, 617 nm emission), and fluorescein (494 nm excitation and 512 nm emission) filters. The fluorescein filter on the Operetta allowed detection of autofluorescent live larvae, which appeared green, while dead larvae appeared bright yellow using the filter for detecting PI staining. Only larvae showing the maximum level of PI fluorescence (designated as 5F) were counted as dead, as we have previously validated 5F larvae to be incapable of infecting rats [35]. The total number of larvae and the number of larvae highly stained with PI were recorded 1 h post-staining and every 24 h for 7 days to follow the progression of L3 mortality. Treatment test results were independently compiled, and results from the replicated tests are provided as an average accompanied by calculations of standard deviation. Statistics (two-sample *t*-tests) were completed using Minitab21.

## 3. Results

### 3.1. Cashido 10 Second Machine Using Direct Delivery of Ozone Gas through a Transfer Pipet

**Figure 1:**
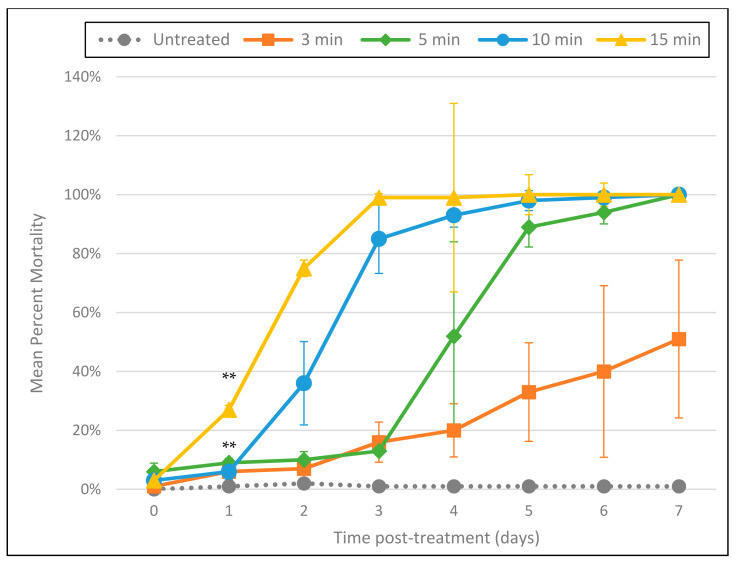
Cashido 10 Second Machine using direct delivery of ozone gas through a transfer pipet at a concentration of approximately 1.5 mg/L. Mean percentage mortality rate per day (as determined by the PI death assay) in response to duration of exposure. All tests were conducted in triplicate. ** All 4 treatments were statistically significant as compared to untreated controls by day 1 (*p* ≤ 0.001).

**Figure 2:**
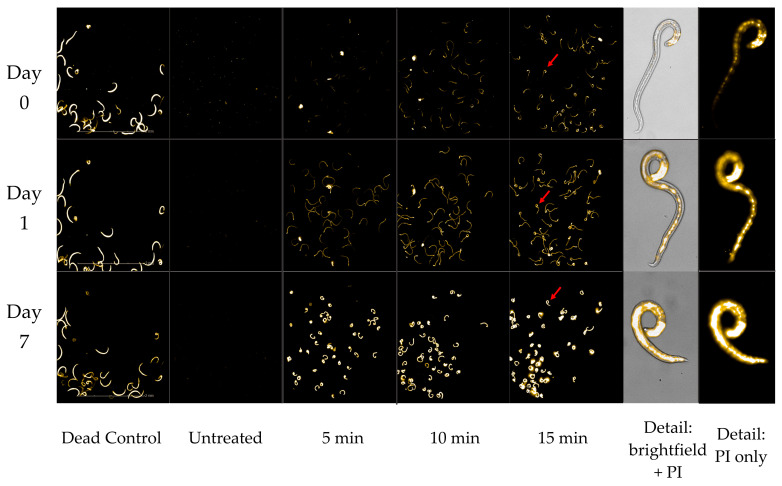
Visual comparison of L3 fluorescence by day to ozone treatment with the Cashido using direct gas delivery. Each row illustrates uniformity of fluorescence in response to ozone for durations of 5, 10, and 15 min (in triplicate), along with a close-up of specific L3 (red arrow) after 15 min exposure.

Using the indigo colorimetric method, the Cashido was found to suffuse ozone into water at a concentration of approximately 1.5 mg/L. Tests (conducted in triplicate) of the Cashido bubbling ozone through a pipet into 5 mL of tap water containing *A. cantonensis* larvae for 3, 5, 10, and 15 min resulted in mortality that followed a clear dose–response relationship to the duration of exposure (Figure 1). Exposure to ozone at all durations resulted in significant mortality by as early as day 1 (*p* ≤ 0.001), and while the 3 min exposure resulted in only 51% mortality by 7 days post-treatment, exposure for 5, 10, and 15 min resulted in 100% mortality by day 7. Interestingly, larvae exposed to ozone for 5, 10, and 15 min all showed a slight but uniform amount of fluorescence on day 0 (approximately 2 h after treatment and 1 h after incubation with PI). The intensity of this fluorescence was also dose-dependent, i.e., brightness was correlated with the duration of exposure (Figure 2). In addition, all day 0 L3 were completely immobile and exhibited a “C” shape frequently associated with larval mortality. By day 1 (approximately 24 h after treatment), the rigid “C” shape had disappeared, but the uniform, dose-dependent fluorescence—while not reaching maximum intensity—was more pronounced. By day 7, all L3 exhibited maximum fluorescence.

### 3.2. Enaly OZX-300 Using Direct Gas Delivery through an Air Stone

**Figure 3:**
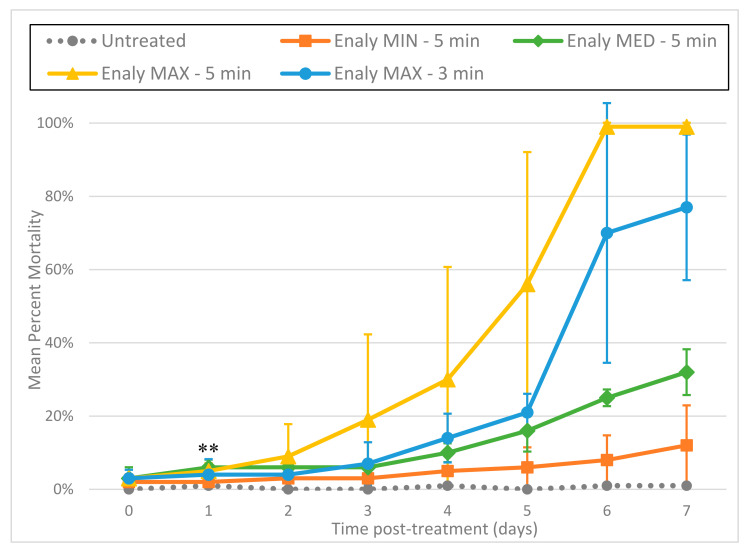
Enaly OZX-300 using direct gas delivery through an air stone at a concentration of approximately 4.5–7.9 mg/L. Comparison of mean percentage mortality rate per day (PI death assay) in response to 3 ozone outputs (minimum, medium, and maximum). All tests were conducted in triplicate. ** All 4 treatments were statistically significant as compared to untreated controls by day 1 (*p* ≤ 0.001).

Using the indigo colorimetric method, we found that the Enaly produced ozone suffused into water at a concentration of approximately 4.5–7.9 mg/L, with concentration corresponding to greater output. This variation in ozone output was reflected in L3 mortality; the Enaly ozone generator showed a clear dose–response relationship between L3 mortality and the setting of the ozone output dial, with the maximum setting associated with the highest mean percentage mortality. Mortality also varied in response to the duration of exposure to ozone, with higher mean percentage mortality at the maximum setting for 5 min compared with 3 min (*p* ≤ 0.001 by day 1) (Figure 3). As with the Cashido, L3 treated with ozone from the Enaly showed a uniformity of fluorescence that correlated with the duration of exposure.

### 3.3. Ultrasonic Cleaners

**Figure 4:**
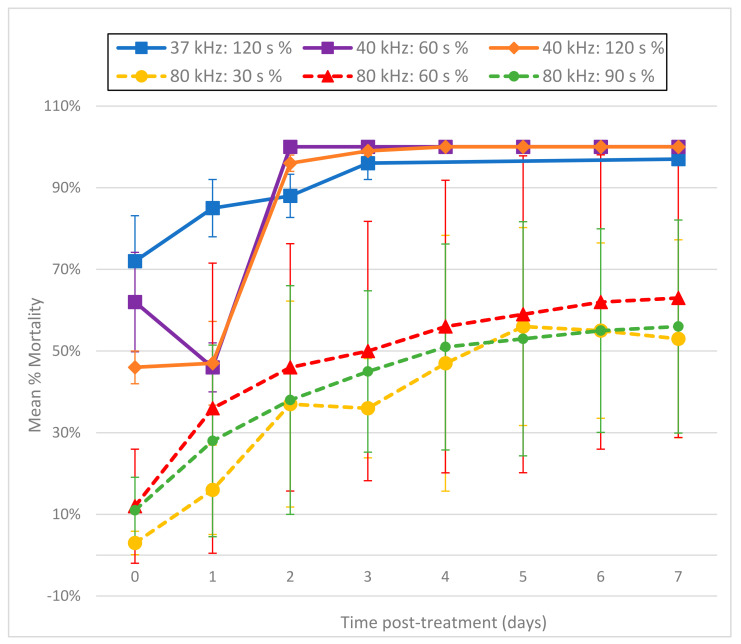
Mean percentage mortality from exposing *A. cantonensis* larvae to Branson 1510R-MT (40 kHz) and Fisherbrand FB-11201 (37 and 80 kHz) ultrasonic cleaners in triplicate. Percentage mortality was normalized by estimating the total number of dead L3 (number of dissolved L3 plus the number of fluorescent L3), and then dividing by the original total number of L3.

**Figure 5:**
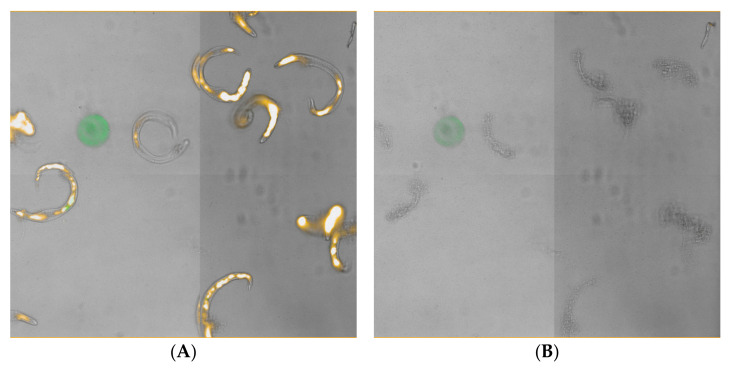
L3 under bright-field and PI illustrating rapid disintegration after ultrasound (2 min at 40 kHz) with the Branson 1510. (**A**) Day 0; note the punctuate fluorescence along the length of the nematode body. (**B**) Day 1, same view; note the loss of fluorescence and general deterioration of the nematode body.

**Figure 6:**
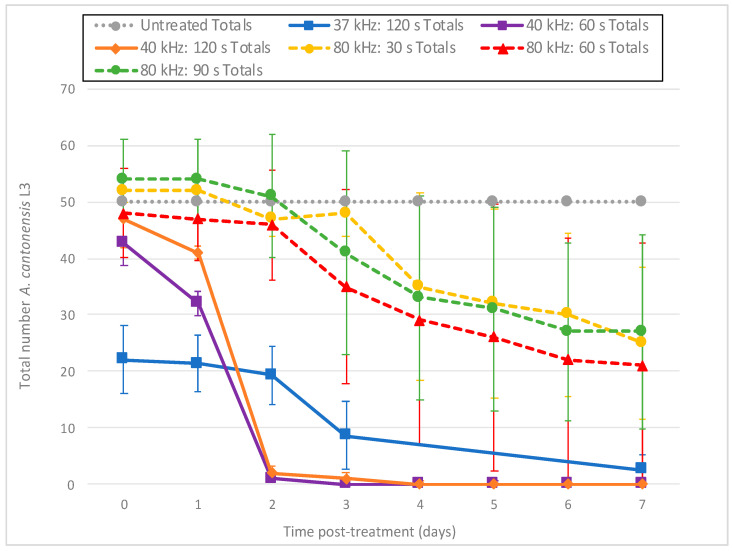
Decline in the total number of *A. cantonensis* L3 per day from exposure to ultrasound at 40 kHz (Branson 1510R-MT) and 37 and 80 kHz (Fisherbrand FB-11201). All tests were conducted in triplicate.

Larvae treated with ultrasound at 37 kHz (Fisherbrand^®^ FB-11201) or 40 kHz (Branson 1510R-MT) exhibited immediate and intense day 0 fluorescence, with 60 s at 40 kHz resulting in 46% mortality within 2 h of treatment based on the PI death assay results (Figure 4). These results suggest that cell membranes were immediately disrupted and allowed rapid entry of PI. This fluorescence appeared in blebs along the length of the individual larvae (Figure 5A). By day 1 (and after), most treated larvae no longer fluoresced, and exhibited progressive disintegration, with microscopic images showing them as indistinct smudges (Figure 5B). Given this rapid dissolution, a count of the total number of nematodes remaining intact per day appeared helpful to provide context to the decreasing number of fluorescent nematodes (Figure 6). Thus, while the number of untreated L3 remained constant throughout the experiment, far fewer L3 were left intact after treatment with the Branson 1510R-MT at 40 kHz for 1 and 2 min, or with the Fisherbrand FB-11201 ultrasonic cleaner at 37 kHz, and those numbers of L3 dropped even further after day 1. By contrast, the effects of ultrasound when using the Fisherbrand^®^ FB-11201 ultrasonic cleaner at the lower intensity of 80 kHz were neither as intense nor as immediate as those at 37 or 40 kHz; indeed, while no live L3 were observed to have survived post-treatment after 37 or 40 kHz, many L3 exhibited movement after treatment at 80 kHz. Therefore, in order to account for the progressive loss of treated L3 in determining mean percentage mortality, the data in Figure 4 are normalized by estimating the number of dead L3 (the number of dissolved L3 plus the number of fluorescent L3), and then dividing by the original total number of L3.

### 3.4. Ozone with Ultrasound

**Figure 7:**
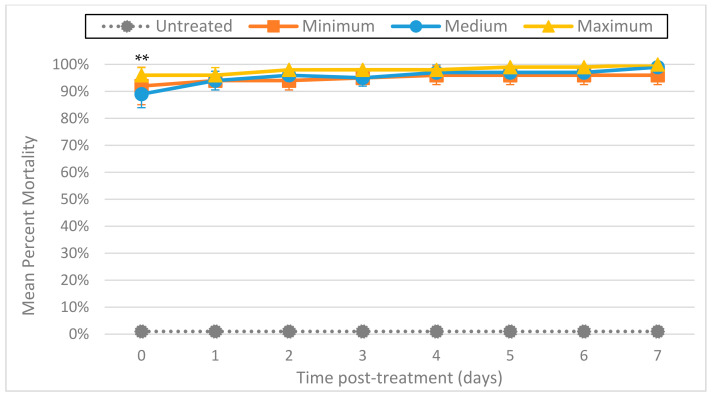
Mean percentage mortality from sonicating *A. cantonensis* larvae at 40 kHz with the Branson 1510 ultrasonic cleaner for 1 min while simultaneously exposing them to ozone for 5 min from the Enaly OZX-300 at 3 different outputs (minimum, medium, and maximum). All tests were conducted in triplicate. Percentage mortality was normalized by adding the number of dissolved L3 to the number of fluorescent L3, then dividing by the original total number of L3. ** All 3 treatments were statistically significant as compared to untreated controls by day 0 (*p* ≤ 0.001).

The effect on larval mortality when using ozone and ultrasound together was dramatic (Figure 7). Regardless of ozone output level (minimum, medium, or maximum), larval destruction was immediate, with all output levels showing a minimum of 89% normalized mean percentage mortality on day 0 (2 h post-treatment) (*p* ≤ 0.001). In addition, microscopic inspection of L3 remaining after combined treatment showed no movement at any time.

## 4. Discussion

Most cases of angiostrongyliasis in the United States, including Hawai‘i, are thought to result from the accidental ingestion of *A. cantonensis* L3 via fresh produce [6], yet there has been relatively little attention paid to developing methods of reducing such transmission. We recently reported on our tests of a range of wash solutions for their efficacy in killing *A. cantonensis* L3, with the goal of reducing accidental infection [15]. Out of the over 40 different treatments that we tested, we found only 8 solutions capable of killing more than 50% of L3 by seven days post-treatment. Of these, two were oxidizers: common household bleach (sodium hypochlorite), and chlorine dioxide. However, the use of these treatments has raised concern due to their combining with organic compounds to form halogenated byproducts with carcinogenic potential, along with their tendency to alter the taste and odor of treated food products [39]. Ozone, which has an oxidation potential even greater than sodium hypochlorite bleach or chlorine dioxide, has the advantage of rapidly decomposing to oxygen, leaving no residue. In addition, ozone generators have become widely available and relatively affordable consumer items, sold as air purifiers and for sanitizing drinking water and food. For all of these reasons, we were interested in testing the efficacy of ozone against *A. cantonensis* third-stage larvae.

Consumer-grade ozone generators designed for food sanitation can be divided between those that suffuse ozone gas into flowing tap water used for rinsing produce, versus those devices that inject ozone gas into a vessel containing water and produce. We tested two consumer-grade ozone generators—the Cashido “10 Second Machine” and the Enaly OZX-300—and of these two, the Cashido was capable of both modes of operation. After running the Cashido-ozonated water into tubes of larvae for 5, 15, and 30 min, we found no evidence of mortality regardless of the duration of exposure. This complete lack of efficacy contrasted sharply with the larvicidal effects gained by injecting ozone gas directly into water containing larvae. Alexopoulos et al. [40] found a similar discrepancy in microbial count between rinsing lettuce in ozone-saturated water versus washing in water with active ozone bubbling, and suggested that this might be due to the more thorough dispersion of ozone into the solution by bubbling. A similar pattern was found in a study of pathogenic *E. coli* counts on apples, which they argued could be due to ozone concentrations being higher at the gas–liquid interface of bubbles than in the surrounding water [19]. Indeed, while ozone is more soluble in water than oxygen, it has a lower partial pressure, and is unstable in water at ambient temperature, making it difficult to obtain high concentrations in solution [17]. Finally, because our experimental design made it impossible to run ozonated rinse water continually over larvae without washing them away, larvae were only incubated in ozone-suffused water for successive 5 min intervals. Since ozone is less stable in the aqueous than the gaseous phase [41], the larvae in our experiment may have received reduced exposure to ozone. Nevertheless, the almost complete lack of mortality strongly suggests that rinsing produce with ozone-suffused water is an insufficient means of eliminating *A. cantonensis* from fresh produce.

By contrast, injecting ozone gas into water containing larvae via either the Cashido or the Enaly generator resulted in over 98% mortality by day 7 post-treatment. Such high mortality is all the more impressive in that it resulted from very little exposure time. Treatments found from previous testing to be the most efficacious at killing L3—such as bleach, chlorine dioxide, various alkaline solutions, the surfactant DBSA, etc. [15]—generally required 60 min of exposure, while ozone produced the same levels of mortality after only 5 min of exposure. Mortality from ozone exposure was consistently dose-dependent, with death rates increasing with the duration of exposure or generator output.

Ozone also appears to differ from previously tested treatments in inducing a degree of uniformity in fluorescence among the L3 treated with ozone and then tested with our PI-based death assay. While we note in our validation of the PI-based death assay that “larvae may show varying levels of PI fluorescence over time as they die” [35], in our tests of ozone, the majority of L3 in each treatment generally showed the same amount of fluorescence, with all showing the same degree of increase each day. Early research into the use of ozone as a bactericide reported an “all-or-none” effect in which ozone inactivation did not take effect until a critical concentration of ozone was reached, after which total inactivation occurred [27,42]. This was explained as representing “that quantity of ozone necessary to produce a detectable residue in suspension” [43]. Thus, the uniformity we note may be another manifestation of the ozone saturation level in the water containing the L3. The significance of this gradual but uniform progression of fluorescence in L3 exposed to ozone is that judging lethality becomes problematic, and might even mean that the mortality percentages listed for day 1—and even day 0—could be underestimations, particularly as we noted the immediate cessation of movement among L3 in all tests of ozone. Alternatively, such low but uniform fluorescence might indicate a degree of injury to the nematodes, resulting in reduced infectivity. Such questions await further testing in animal models.

We tested the effects of sonication at both 37 and 40 kHz, as well as at the lower intensity of 80 kHz, and found that 37 and 40 kHz were both fast and effective at killing *A. cantonensis* L3. A striking aspect of ultrasound was the speed at which it induced larval death, with mortality rates on day 0 (2 h post-treatment) as high as 72% after only 2 min of exposure to 37 kHz (Figure 4). However, such mortality results were quite variable (±11.14 standard deviation, *p* ≤ 0.001), with some tests with ultrasound leaving small numbers of L3 still motile. This variation was probably due to spatial variations in ultrasound intensity within the water bath, as rates of mortality appeared to be dependent upon the placement of the tube of larvae within the bath of the ultrasonic cleaner.

Tests in which larvae were subjected to both ultrasound and ozone simultaneously showed dramatic increases in mortality, suggesting that the two treatments are highly synergistic. Indeed, while ozone exposure was for 5 min, sonication was limited to only 1 min, simply because the application of longer durations of ultrasound left no intact larvae available to count. Thus, comparing the mortality induced by ozone vs. ultrasound vs. the two treatments combined, their combined use clearly resulted in higher initial mortality than either treatment individually. Moreover, as the efficacy of ultrasound appears prone to variation (as described above), combining ultrasound with ozone would seem to take advantage of both methods’ strengths. Further research should be conducted to determine the optimal duration of ozone and ultrasound exposure, so as to maximize lethality while minimizing the hazardous effects of ozone.

Given ozone’s high reactivity, significant safety issues must be considered before contemplating its use in rendering food safe from *A. cantonensis*. The primary risks are to the nasal mucosa, respiratory tissue, and eyes. The U.S. Occupational Safety and Health Administration (OSHA) guidelines call for exposure limits of 0.1 ppm for 8 h per day [44]. Thus, any ozone system under consideration for consumer use must include some means of containing ozone during treatment and destroying any excess—for example, by passage through a column of activated carbon.

## 5. Conclusions

We evaluated both ozone gas and ultrasound for their efficacy in killing the infectious third-stage larvae (L3) of *A. cantonensis* using commercially available ozone generators and ultrasonic cleaners (two of each). We found ozone to be capable of achieving 100% L3 mortality, albeit at varying time spans depending upon ozone concentration and duration of exposure. Sonication applied at frequencies of 37–40 kHz produced even more rapid mortality, with mortality rates as high as 72% after only 2 min of treatment, but with results that were quite variable. The combination of both ozone and ultrasound was most efficient, resulting in up to 89% normalized mean mortality within 2 h of treatment. While no minimal infectious dose for angiostrongyliasis has been determined [45], there does appear to be evidence that disease severity correlates with worm burden [46,47,48]. Given the ability of ozone and ultrasound to engender significant mortality in *A. cantonensis*, overall, these technologies would appear to have the potential to provide a safer and more convenient means of reducing the risk of *A. cantonensis* transmission via accidental consumption. Indeed, both ozone and ultrasound technologies are already available as “fruit and vegetable cleaners” for home use and in commercial food sanitation. By supplementing close examination and rinsing of produce with water—the only current means of preventing infection—such cleaners could play an important role in areas of the world where *A. cantonensis* is endemic. However, many questions remain. Our in vitro assay of L3 mortality needs validation in an animal model, with a particular need for further research on the ozone concentrations and ultrasound durations necessary for optimal killing efficiency. Such parameters, in turn, need to be examined as to their effects on other aspects of food safety, such as human exposure to ozone gas, as well as their impacts on food palatability and storage life.

## Data Availability

All data supporting the reported results are provided in the manuscript.
